# Study protocol for reducing childbirth fear: a midwife-led psycho-education intervention

**DOI:** 10.1186/1471-2393-13-190

**Published:** 2013-10-20

**Authors:** Jennifer Fenwick, Jenny Gamble, Debra K Creedy, Anne Buist, Erika Turkstra, Anne Sneddon, Paul A Scuffham, Elsa L Ryding, Vivian Jarrett, Jocelyn Toohill

**Affiliations:** 1 School of Nursing & Midwifery, Griffith University, Logan Campus, University Drive, Meadowbrook, QLD 4131, Australia; 2 Griffith Health Institute Griffith University, University of Queensland, St Lucia, Brisbane, QLD 4072, Australia; 3 University of Melbourne, Grattan Street, Parkville, VIC 3010, Australia; 4 Gold Coast Hospital, Griffith University, Parklands Drive, Southport, Qld, Australia; 5 Department of Women’s & Children’s Health, Karolinska Institute, Stockholm, Sweden

**Keywords:** Pregnancy, Childbirth fear, Psycho-education, Midwife-led counselling, Randomised controlled trial, Postnatal depression, Decisional conflict, EQ-5D, Self-efficacy

## Abstract

**Background:**

Childbirth fear has received considerable attention in Scandinavian countries, and the United Kingdom, but not in Australia. For first-time mothers, fear is often linked to a perceived lack of control and disbelief in the body’s ability to give birth safely, whereas multiparous women may be fearful as a result of previous negative and/or traumatic birth experiences. There have been few well-designed intervention studies that test interventions to address women’s childbirth fear, support normal birth, and diminish the possibility of a negative birth experience.

**Methods/design:**

Pregnant women in their second trimester of pregnancy will be recruited and screened from antenatal clinics in Queensland, Australia. Women reporting high childbirth fear will be randomly allocated to the intervention or control group. The psycho-educational intervention is offered by midwives over the telephone at 24 and 34 weeks of pregnancy. The intervention aims to review birth expectations, work through distressing elements of childbirth, discuss strategies to develop support networks, affirm that negative childbirth events can be managed and develop a birth plan. Women in the control group will receive standard care offered by the public funded maternity services in Australia. All women will receive an information booklet on childbirth choices. Data will be collected at recruitment during the second trimester, 36 weeks of pregnancy, and 4–6 weeks after birth.

**Discussion:**

This study aims to test the efficacy of a brief, midwife-led psycho-education counselling (known as BELIEF: Birth Emotions - Looking to Improve Expectant Fear) to reduce women’s childbirth fear. 1) Relative to controls, women receiving BELIEF will report lower levels of childbirth fear at term; 2) less decisional conflict; 3) less depressive symptoms; 4) better childbirth self-efficacy; and 5) improved health and obstetric outcomes.

**Trial registration:**

Australian New Zealand Controlled Trials Registry ACTRN12612000526875.

## Background

The prevalence of fear associated with childbirth is around 20% with approximately 6-10% of women experiencing intense fear of labour and birth that is dysfunctional or disabling [1]. A further 13% of non-pregnant women are fearful enough of childbirth to postpone or avoid pregnancy [2]. Childbirth fear has received considerable attention in Scandinavian countries, and the UK [3-6] but not in Australia. Our previous qualitative and quantitative work suggests that levels of childbirth fear are significant. For example, in our large qualitative study (n = 202) approximately 22% of mothers used words such as *terrifying* and *petrifying* to describe their expectations of birth [7,8]. Our subsequent survey of 400 pregnant women using the Expectations and Experiences Questionnaire (WDEQ) [9] found that nearly 50% of women reported moderate fear and 26% reported intense fear of childbirth [10]. These figures are much higher than those reported in Scandinavian studies.

While most studies have found nulliparous women to be more fearful than multiparous women, the reverse has also been demonstrated [11-13]. While this fear is commonly associated with concern for the baby it is also related to uncertainly about the unknown [14]. For some first-time pregnant women a perceived lack of control mixed with a disbelief in the body’s ability to give birth safely becomes overwhelming and generates extreme fear; referred to as primary childbirth fear [11,15]. When multiparous women admit fear it is often a result of a previous negative and/or traumatic birth experience [13]. This is described as secondary fear or tocophobia [16].

Evidence suggests that childbirth fear, confidence (self-efficacy) and a sense of control are closely linked [3,13]. Lowe [4] found that confidence for labour was significantly higher in women reporting low fear. Conversely, women with high fear reported more learned helplessness, belief in chance rather than self-control, and attributed control to powerful others. In another study women reporting intense childbirth fear were worried about their performance in labour and their bodies’ abilities to birth [17]. Their concerns translated into low expectations of positive outcomes and their ability to cope with labour. Other factors influencing women’s fear relate to the context or environment in which women birth and their interactions with health care professionals. In Sjogren’s [17] work, the most common reason for fear was lack of trust in maternity staff and the system (73%); a factor that has been replicated across cultures [18-20].

### Existing interventions to assist women fearful of birth

Approaches to assist women reporting childbirth fear have been pioneered in Scandinavian countries. In Sweden many obstetric departments have established qualified teams to support women who identify as highly fearful of birth. Care is provided by midwives with mental health qualifications and supported by psychologists, psychiatrists and obstetric staff. Treatment consists of 2–4 visits with partners, relaxation techniques, a visit to the labour ward and development of an individualized birth plan [18]. Fearful women who initially wished to be delivered by caesarean section (CS) were less inclined to do so after counselling [19-22]. In comparative studies, an evaluation of midwife-led counselling with 53 fearful women compared to matched controls produced positive results [19], however, Sjorgren et al. [23] found no difference between women treated for fear and non-fearful controls. Recently, Finnish researchers tested the effectiveness of 5 group sessions facilitated by a psychologist with first time pregnant women with intense fear and requesting a CS. The control group received standard treatment (2 visits with an obstetrician trained in psychotherapy). This study produced positive results with 82% of women changing their preference for a CS as opposed to 67% in the standard treatment group [24]. The relative lack of well-designed intervention studies suggests that it is timely to test a clinically relevant and proven midwife-led psycho-education intervention that has the potential to address women’s childbirth fear, support normal birth, and diminish the possibility of a negative birth experience.

### Aims

To test the efficacy of a psycho-education counselling intervention offered by midwives to address women’s fear of childbirth.

## Methods/design

Approximately 1200 women will be recruited and screened during the 2nd trimester of their pregnancy. Women reporting high levels of fear will be randomly allocated to BELIEF or control group. Women in the intervention group will receive 2 counselling sessions at approximately 24 and 34 weeks of pregnancy. Women in the control group will receive standard care. All women will be given the evidence-based consumer resource “Choosing how to birth your baby” developed by the Queensland Centre for Mothers and Babies. Both groups will be followed up to 4 to 6 weeks postpartum.

Providing a control group that receives the same written evidence-based information establishes whether the counselling intervention accounts for the results. The intervention is targeted to those women reporting high levels of childbirth fear. Using registered midwives, who will be trained and supervised to deliver BELIEF, maximises the translation of research findings into practice.

### Hypotheses

1) Relative to controls, women receiving BELIEF will report lower levels of childbirth fear at term; 2) less decisional conflict; 3) less depressive symptoms; 4) better childbirth self-efficacy; and 5) improved health and obstetric outcomes.

### Outcome measures

The primary outcome measure is a reduction in childbirth fear according to the Wijma Delivery Expectancy/Experience Questionnaire. Secondary outcome measures include the Decisional Conflict Scale, Edinburgh Postnatal Depression Scale, Childbirth Self-Efficacy Inventory, EuroQol 5 dimensional scale, mode of birth, and health service use.

### Setting

Gold Coast, Logan and Redland Hospitals are large facilities in South-East Queensland, Australia with approximately 8,500 births per year.

### Sample and sample size

Pregnant women in their 2nd trimester (12–24 wks) will be invited to participate. Using a significance level of 5%, power of 80%, and a two-tailed test, a sample of 150 participants in each group is required (recruitment approx n = 1200 women prior to randomisation). The sample size calculation assumes that around 20% of women at recruitment (approximately 20 weeks gestation) will report high levels of fear [10] and consent to participate. Calculations for recruitment allowing for loss to follow-up are based on a formula derived from our previous work. Expected number of eligible women per month = 466. Some women may be missed or lost to follow-up and we expect an attrition rate of 30%.

### Inclusion criteria

All women attending the antenatal clinics of participating sites in the second trimester of pregnancy will be invited to participate. The women must be able to communicate sufficiently to discuss their concerns about birth and be 16 years or older. Women scoring high for childbirth fear (WDEQ-A ≥66) will be randomized to either the control or intervention group.

### Exclusion criteria

If after recruitment women come to expect a perinatal death (e.g. congenital abnormality incompatible with life) or stillbirth they will be given an opportunity to withdraw. If they opt to continue they will be offered counselling support and receive newsletters but not included in the analysis. Follow-up questionnaires containing parenting questions will not be administered.

### Recruitment protocol & randomisation procedure

The research midwives will follow a recruitment protocol which adheres to the National Health and Medical Research Council ethical guidelines for human research. Clinic staff will be briefed on the project and invite eligible women to speak to the research midwife attending the antenatal clinic. After obtaining informed consent, women will be invited to provide contact details and complete a questionnaire. Questionnaire responses will be scored. Within 72 hours, women who score ≥66 on the WDEQ-A will be contacted and with their permission randomised into the intervention or control group using a centralised block randomisation with stratification for different sites and parity with a 1:1 allocation. The research-midwife will access the web-based randomisation service after gaining participant’s written consent and not blinded to the woman’s allocation.

Women receiving the BELIEF intervention will be offered a convenient time for the first of two telephone counselling sessions. Participants will also be provided with a contact telephone number (available 9 am-8 pm) if they require additional support. Details of this contact will be logged.

All women will be monitored for psychological safety by research staff. We have developed a risk protocol that clearly sets out referral pathways. Participation is not expected to disadvantage any women. Women receiving the intervention are expected to have reduced psychological distress and women in the control group will be monitored and receive scheduled measurement of their psychological well-being. Women will be referred to pre-arranged counselling services if necessary and excluded from the study but included in the intention-to-treat analysis. Although the research midwife will not be blinded to group allocation, follow-up assessment will be conducted by an independent research assistant not involved in the intervention. Additionally analysis will be blinded.

### Measures

*Demographic, Reproductive & Health Questionnaire* seeks personal information, such as age, educational level, income, ethnicity and marital status. Obstetric details will include relevant history (e.g. parity and miscarriage) and birth plans for the current pregnancy (including preferred mode of birth).

*Wijma Delivery Expectancy/Experience Questionnaire (W-DEQ)* is a 33-item, 6-point Likert scale questionnaire that measures women’s fear of childbirth. Items refer to expectations and experiences before birth (version A) and after birth (version B). It has good psychometric properties with a high internal consistency and split-half reliability (> 0.94 before and > 0.87 after birth) with nulliparous and multiparous women [9,13]. Internal reliability in a population of Australian pregnant women was high with a Cronbach’s alpha of 0.92 [10].

*Edinburgh Postnatal Depression Scale (EPDS)* is a 10 item well validated self-report questionnaire designed to screen for depression [25]. It has a split-half reliability of 0.88 and standardised alpha coefficient of 0.87. Range of scores is from 0–30 with postnatal scores above 12 indicative of probable depression [25].

*Childbirth Self-Efficacy Inventory (CBSEI)* is a 62-item scale [26] that requires responses on a 10-point Likert scale. High scores indicate stronger self-efficacy or outcome expectancy for birth. The CBSEI has been validated for use in the Australian birthing population and reported reliability coefficients for all four subscales are above 0.90 [27].

*Decisional Conflict Scale (DCS)* is a 16 item scale, which investigates factors that compromise or facilitate effective decision making [28] and has been widely used in decision aid research including VBAC within the Australian context [29]. Internal consistency coefficients ranged from 0.78 to 0.92 and discriminated significantly (p < 0.001) between those who had strong intentions either to accept or decline a method of care to those who were uncertain [28].

*EuroQol (EQ-5D)* is a 5-item self-report scale that measures health-related quality of life using self-rating of current health state across the 5 dimensions of mobility, self-care, usual activities, pain/discomfort, and anxiety/depression [30]. Each item assesses 3 levels of severity ranging from 1 = no difficulty to 3 = great difficulty. Higher item scores indicate poorer health-related quality of life. Test-retest reliability of the EQ-5D at 10 months was .90. All measures have been previously used with the Australian childbearing population.

### Postnatal questionnaire – 4–6 weeks postpartum

Data will be collected on maternal and neonatal outcomes including mode of birth, use of interventions such as induction, anaesthesia (e.g. epidural) and neonatal outcomes. Participants will be asked about their experience of labour, birth, and postnatal care, involvement in decision making, satisfaction with care, and future birth plans. This questionnaire will be adapted from the QCMB Survey – Having a Baby in Queensland [31]. This information will enable comparison with the birthing population in Queensland.

*Parenting Sense of Confidence and Satisfaction (PSOC - 12 items)* measures self-efficacy and satisfaction derived from parenting and is commonly used [32]. Only the self-efficacy factor (7 items, 6-point Likert scale) will be used. This factor has good internal consistency (α .76) (62) with higher scores indicating stronger self-efficacy.

*Health service use (HSU)* The use of health services (GP visits, emergency department attendances and hospital admissions) by both the mother and the baby will be collected retrospectively. Health service use by the mother and baby will be obtained from Medicare Australia for the period from 20 weeks gestation to 4 to 6 weeks follow-up. The diagnosis and Australian Refined Diagnostic Related Group (AR-DRG) will be collected for all reported admissions to hospital for the same period. We have used these scales previously with the Australian childbearing population. The questionnaires take approximately 40 minutes to complete.

### Consent

Eligible women will be given written information outlining the study purpose, invited to ask questions, and be able discuss their participation with family or care providers. Women agreeable to participation will provide written consent. Women wishing to consider their involvement will be followed up by telephone within 48 hours of their booking appointment. Participants will be advised they can withdraw from the study at any time without effect to their care but will not receive payment for participation. Women will also be provided with contact details for the research team, Griffith University and participating Hospital research ethics committees should they wish to discuss any concerns regarding conduct of the study.

### Control group

Women in the control group will receive standard care. Standard care includes booking care with a hospital midwife. At this time, the woman’s history and medical notes are reviewed, assessment and discussion of birth options provided, and antenatal options of care within a midwifery model, general practitioner shared care in the community or obstetric care in the hospital is offered. All women regardless of model of care have a hospital appointment at 36 weeks of pregnancy when a birth plan is usually confirmed. All women receive inpatient intrapartum and postnatal care from rostered hospital staff. Women in the control group will receive an information booklet developed by the Queensland Centre for Mothers & Babies on preparing for childbirth.

### Intervention

#### Counselling

A brief midwife-led counselling intervention that does not require the midwife to have advanced psychotherapeutic qualifications has been developed and tested with postnatal women and found to reduce postnatal emotional distress [33]. The intervention will occur by telephone. The intervention will be provided between 24 and 34 weeks gestation and support women to examine the origin of childbirth fear, reconcile any impact from a previous birth experience, be informed of their birth options and develop strategies for a positive birth experience regardless of the ultimate mode of birth. The counselling intervention 'Promoting Resilience in Mothers’ Emotions’ (PRIME) [34] will be adapted for use in this study for two reasons; the benefits of the counselling approach has applicability to the target population and it is reproducible. This counselling approach has demonstrated statistically significant sustained improvement in women’s emotional health and confirmed that women value the opportunity to talk about their birth experience and to have these experiences validated [35]. While this specific counselling intervention has not been tested in the antenatal period, it has been found in the postnatal period to reduce symptoms of trauma, depression, stress, and feelings of self blame [34]. The notion of self-blame has implications for women in the current study who may have unresolved emotional or psychological effects from a previous birth experience and impact their confidence to birth. Previous stressful birth events may be heightened in a next pregnancy impacting women’s decision making about birth [35]. Responding to women’s concerns by way of a psycho-education intervention, it is proposed that women will be more confident and be better informed to pursue vaginal birth where this is an option for them.

The key elements of BELIEF are outlined in Table 1. The focus of the midwife-led counselling intervention will be on reviewing current expectations and feelings such as fear of childbirth. The midwife researcher’s detailed knowledge of maternity services and childbearing will assist women to become confident in their own ability to birth. The intervention aims to support the expression of feelings and provide a framework for women to identify and work through distressing elements of childbirth. The intervention develops women’s individual situational supports for the present and near future, affirming that negative events during childbirth can be managed and developing a simple plan for achieving this. This combination of strategies diminishes emotional distress, builds constructive coping mechanisms and facilitates recovery.

**Table 1:** **Key elements of the PRIME counselling intervention on which BELIEF is based**[35]

**Strategy**	**Key elements of intervention**
Therapeutic connection between midwife and woman.	Show kindness; affirm competence of the woman, simple non-threatening open questions about the birth, attentive listening and acceptance of the woman’s perspective.
Accept and work with women’s perceptions.	Prompt the woman to tell her own story, listen with encouragement but not interruption.
Support the expression of feelings.	Encourage expressions of feelings by open questions, actively listening, reflecting back the woman’s concerns.
Filling in the missing pieces	Clarify misunderstandings, offer information, answer questions realistically and factually, ask questions about key aspects to check understanding. Do not defend or justify care provided.
Connect the event with emotions and behaviours.	Ask questions to determine if the woman is connecting current emotions and behaviours with prior birthexperiences. Acknowledge and validate emotions. Gently challenge and counter distorted thinking such asself-blame and a sense of inadequacy, Encourage the woman to see that inappropriate or hasty decisionsmay be a reaction to the birth.
Review prior labour management.	Ask if the woman felt anything should have been done differently during labour. Offer new or more generous or accurate perceptions of the event. Realistically postulate how certain courses of action may have resulted in a more positive outcome. Acknowledge uncertainty.
Enhance social support.	Initiate discussion about existing support networks. Talk about ways to receive additional emotional support. Help the woman understand that her usual support people may be struggling with their own issues.
Reinforce positive approaches to coping.	Reinforce comments by women that reflect a clearer understanding of the situation, plan for the way forward or outline positive action to overcome distress. Counter oblique defeatist statements.
Explore solutions.	Support women to explore and decide upon potential solutions, e.g., support group(s), further one-to-one counselling, seeking specific information.

#### Education information

The provision of evidenced-based information for women is considered critical to their informed decision making [36]. Women’s values and experiences must be acknowledged when considering birth options [37] and psychosocial factors are significant to women’s decision making [38]. Additionally evidenced-based information is not easily discriminated from professional or organisationally defined practices as maternity staff are often time poor to discuss these differences [39,40].

Despite the antenatal period being an opportune time to discuss women’s birth preferences and provide information and evidence specific to their personal circumstances, frequently only general advice is provided. Women are seldom afforded the opportunity to explore the events that transpired in their previous pregnancy and birth or to clarify strategies that may assist in achieving a different or hoped for outcome in the current pregnancy with their health care provider [29].

Therefore two avenues will be used to provide education. Firstly, concurrent with the above counselling intervention strategies (Table 1), evidence based information will be integrated into the counselling session that is specific to the woman’s situation. Education will be integrated into 'filling in the missing pieces’ , 'reviewing the labour management’ , 'enhancing social support’ , 'reinforcing positive approaches to coping’ and 'exploring solutions’. The psycho-education sessions will be responsive to the woman’s needs and appropriate integration of information will occur opportunistically and be consistent with the evidence based written information. Women will be encouraged to plan for a positive birth and can ask to clarify their birth options with care providers. All participating women will receive a copy of an evidence-based resource on childbirth choices.

#### Support

Women in the intervention group will also have access to their named research midwife or the project manager by telephone throughout the study period; however the research midwives will not initiate contact with participants outside the scheduled intervention or data collection times. Support is to clarify or discuss any considerations for the woman around her forthcoming birth. Women will be advised that contact for support with the research midwife can occur within 9 am to 8 pm. Participant initiated telephone calls will be diarised to include the woman’s gestation, issues raised, the content of responses provided, and duration of calls. The importance of an accessible source of information for women to talk about their upcoming birth has recently been demonstrated in the uptake of a telephone service within a tertiary setting [41].

Although the majority of women prefer a vaginal birth, recruitment and intervention close to the woman’s first antenatal hospital appointment may encourage women to consider their options earlier. However a strategy to support women to negotiate the maternity system across the pregnancy is required. In one study women planned a vaginal birth at 36 weeks gestation but Shorten et al. [29] identified that organisational culture impacted women securing their preferred mode of birth. Consistent support may minimise the likelihood of women consenting to a birth option that differs from their preference, and this might be particularly important given it has been reported that anxiety in women is higher around 37 weeks [42]. Therefore, along with access to ongoing telephone support, a second counselling intervention will occur after 34 weeks and prior to the routine36 week antenatal appointment with a medical practitioner. This session will consolidate strategies women may like to adopt in order to clarify, or secure, their preferred mode of birth at time of next appointment. While the purpose of the study is to reduce fear, support women’s decision making and subsequently improve vaginal birth rates, the intervention is most importantly to assist women to develop strategies for a positive birth experience regardless of the ultimate mode of birth.

### Midwife training for the psycho-education counselling intervention

The training program includes two intensive 4 hour workshops, written manuals, web-based resources, a DVD of counselling skill vignettes related to the counselling framework, personal supervision of undertaking the counselling, and assessment criteria for competence. The quality and effectiveness of the counselling training will be evaluated through supervision processes, integrity checks, regular group meetings with midwives, a client satisfaction survey as well as research measures of clinical effectiveness [34,35]. Maintenance of the midwife researcher’s counselling competency will be monitored throughout the study.

### Integrity/adherence to counselling intervention protocol

Each of the first five telephone interviews for every midwife counsellor will be independently reviewed and rated for adherence to the counselling strategy.

### Quality and safety

For consistency of the intervention and to determine ongoing competency of the midwives' counselling skills all participants in the intervention group will be asked for permission and their written consent to audio tape the intervention. Members of the research team as well as the project manager (herself skilled in the intervention) will review the counselling intervention for quality and adherence to the intervention.

Participant recruitment and retention will be reviewed monthly and monitored against study timeframes, the intention of the research, for feedback from participants, and to monitor outcome indicators along with any adverse events. A risk assessment will be observed with any distressed women referred via the hospital’s usual processes for appropriate psychosocial intervention in consultation with the obstetric and perinatal mental health team, with the woman withdrawn from the study.

### Data collection

**Figure 1:**
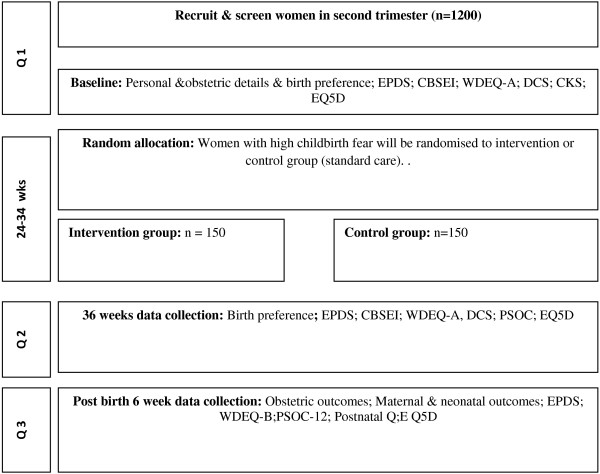
Key points for data collection.

Data collection is shown in the flow diagram (Figure 1). Data will be collected at 3 time points; Recruitment ≈ 20 weeks (T1), 36 weeks gestation, (T2), 4–6 weeks postpartum (T3). Women will be given the option of completing via the post or via telephone. In our previous studies, telephone interviews facilitated a good response rate and were a reliable and accurate way of collecting information. They were also considered preferable by participants given the demands and unpredictability of new motherhood.

### Data management and analysis

The commercial product Remark Office OMR [43] will be used to export raw data from the study tools into the Statistical Package for the Social Sciences (SPSS), minimizing data entry errors. Relationships between categorical variables will be examined using chi-square analyses, between continuous variables Pearson’s product moment correlation test and for relationship between continuous and categorical variables one-way ANOVA of variance. An alpha level of 0.05 will be used for all statistical tests with bonferonni correction where applicable.

A preliminary analysis will examine the pattern of responses to demographic items, and to scales and subscales. The purpose of this analysis is to identify factors that might be influential in determining subscale and scale scores regardless of treatment group. Where necessary, equivalent non-parametric procedures will be used. The issue of missing data owing to participant dropout will be addressed using either multiple imputation or a direct maximum likelihood approach to estimation. These procedures represent the current state of the art in handling missing data in RCTs [44]. In this way, power is maximised by using all available data points in a statistically appropriate manner. Potentially confounding variables such as age, and parity can be entered into the statistical models to adjust for any impact.

Analysis of covariance (ANCOVA) will compare post-treatment scores for the intervention and control groups, with the baseline score as the covariate. Secondary analyses will include a series of independent group t-tests to compare change scores (baseline minus treatment) for the intervention and control groups for other outcomes measures. The chi square statistic will compare percentages of cases in the intervention and control groups who improved, remained the same, or deteriorated.

### Ethical approval

The research protocol has been reviewed and received Human Research Ethics Committee Approval from Queensland Health (HREC/11/QGC/162) and Griffith University (NRS/45/11/HREC).

## Discussion

This proposal addresses the 2011 Maternity Services Special Initiative of the National Health and Medical Research Council in Australia. The protocol tests the effectiveness of an evidenced based midwifery led intervention (BELIEF) designed to improve maternal and perinatal outcomes. The key outcomes are to optimise the mental health of pregnant women, promote normal birth and deliver cost savings to the health care system.

The proposed project evaluates an innovative counselling intervention that integrates evidence and is feasible in clinical practice. First, it uses a tested intervention that combines evidence-based counselling strategies with a comprehensive understanding of the childbearing and new motherhood experiences [35]. Second, it delivers the intervention by telephone thereby improving accessibility, timeliness and flexibility for pregnant women. Third, the intervention does not focus on psychopathology; rather it reinforces and supports women’s resilience, confidence and a sense of competence.

Findings of this study will contribute knowledge and understanding of the antenatal assessment of psychological risk, specifically fear of childbirth, in pregnant women; how to preserve low intervention births; and the provision of effective emotional care in the antenatal period by midwives. The evidence gained from the study will also inform maternity service provision and policy by adding to our understanding of the components of quality maternity care that promote a positive transition to motherhood for Australian women. Addressing childbirth fear and promoting positive attitudes to labour and birth in pregnant women is a critical strategy in preserving the normality of birth and reducing both the primary and repeat caesarean section rate. Achieving this will improve women’s quality of reproductive life, reduce health care costs, improve postpartum outcomes and promote positive child development.

### Timeframe

The study is expected to take 3 years. The first 6 months will be required to hire and train staff. Recruitment will then commence and continue for 12 months (mid Year 2), with final data collection continuing to 4–6 weeks post birth (early year 3). In year 3 we will complete data analysis, reporting and prepare publications and refine resources developed in the project.

## Abbreviations

BELIEF: Birth emotions - looking to improve expectant fear; W-DEQ: Wijma delivery expectancy/experience questionnaire; DCS: Decisional conflict scale; EPDS: Edinburgh postnatal depression scale; CBSEI: Childbirth self-efficacy inventory; EQ-5D: EuroQol 5 dimensional scale; AR-DRG: Australian refined diagnostic related group.

## Competing interests

The authors declare that they have no competing interests.

## Authors’ contributions

JF is the CIA and lead the overall conceptual design and preparation of the study protocol in collaboration with the CI team. JF, DC and JT refined the protocol, drafted the manuscript and co-ordinated revision of the manuscript. JG, ET, DC, AB and AS were involved in the conceptual design of the study and revision of the manuscript. All authors have read and approved the final manuscript.

## Pre-publication history

The pre-publication history for this paper can be accessed here:

http://www.biomedcentral.com/1471-2393/13/190/prepub
